# Mapping home internet activity during COVID-19 lockdown to identify occupation related inequalities

**DOI:** 10.1038/s41598-021-00553-7

**Published:** 2021-10-26

**Authors:** Cameron Zachreson, Erika Martino, Martin Tomko, Freya M. Shearer, Rebecca Bentley, Nicholas Geard

**Affiliations:** 1 School of Computing and Information Systems, The University of Melbourne, Melbourne, Australia; 2 Melbourne School of Population and Global Health, The University of Melbourne, Melbourne, Australia; 3 Faculty of Engineering and Information Technology, The University of Melbourne, Melbourne, Australia; 4 Department of Infectious Diseases, Melbourne Medical School, The University of Melbourne, Melbourne, Australia

**Keywords:** Environmental social sciences, Diseases, Risk factors

## Abstract

During the COVID-19 pandemic, evidence has accumulated that movement restrictions enacted to combat virus spread produce disparate consequences along socioeconomic lines. We investigate the hypothesis that people engaged in financially secure employment are better able to adhere to mobility restrictions, due to occupational factors that link the capacity for flexible work arrangements to income security. We use high-resolution spatial data on household internet traffic as a surrogate for adaptation to home-based work, together with the geographical clustering of occupation types, to investigate the relationship between occupational factors and increased internet traffic during work hours under lockdown in two Australian cities. By testing our hypothesis based on the observed trends, and exploring demographic factors associated with divergences from our hypothesis, we are left with a picture of unequal impact dominated by two major influences: the types of occupations in which people are engaged, and the composition of households and families. During lockdown, increased internet traffic was correlated with income security and, when school activity was conducted remotely, to the proportion of families with children. Our findings suggest that response planning and provision of social and economic support for residents within lockdown areas should explicitly account for income security and household structure. Overall, the results we present contribute to the emerging picture of the impacts of COVID-19 on human behaviour, and will help policy makers to understand the balance between public health and social impact in making decisions about mitigation policies.

## Introduction

Globally, the COVID-19 pandemic has changed the ways in which people live and work. Governments have used various mitigation strategies to stem the spread of infection by restricting mobility and limiting the rate of physical interaction between people^1^. In Australia, these strategies have included limiting the size of gatherings, restricting travel across state borders, closing workplaces, and encouraging working and schooling from home. To help ensure compliance with these policies, and to dampen their short-term economic impact, the Australian government in early 2020 implemented social assistance programs, including the temporary COVID Supplementary payment for the unemployed (JobSeeker), the JobKeeper wage subsidy, early access to superannuation (retirement) accounts, and targeted support for severely affected sectors^2,3^. However, these measures cannot be maintained indefinitely, and the protracted COVID-19 pandemic may outlast Australia’s ability to continue providing the high levels of economic support that helped ensure compliance with restrictions. The broad aim of the present study is to quantify and map socioeconomic factors that are likely to determine current and future adherence to mobility restrictions. Detailed knowledge of the geographic distribution of these factors will assist when predicting the social impact of targeted mitigation strategies during future outbreaks of COVID-19, or subsequent pandemics.

Broadly, we expect a reduced effectiveness and increased economic impact of such mobility restrictions where there is a concentration of people who cannot adapt to home-based work. We know, for example, that people in factory-based occupations, hospitality, and essential services are unable to work from home^4–7^. The confluence of occupational and financial constraints place many such workers at a greater risk of exposure to infectious disease, either through the occupational hazard of close social interactions, or because without adequate leave or income entitlements, they have a limited ability to remain at home when unwell^6,8,9^. This conflict between household economic needs and public health orders to stay at home is problematic for the success of such mitigation strategies.

COVID-19 has driven a general economic contraction brought about through concerted reductions in consumer, recreational, and occupational activity^10–12^. Impact disparities can be explained (at least partially) by examining the distribution of employment types within and between subpopulations. Those who can perform their work requirements at home from a computer with internet access have experienced less-severe economic impact^13–18^. By engaging in written communication, and replacing face-to-face interactions with online video conferencing, work-related tasks are likely to result in measurable increases to home internet traffic. Population-level data on home internet usage may therefore provide a useful complement to the widely available mobility data typically used to monitor and model the real-time effects of COVID-19 and the restrictions associated with mitigation measures^16,17,19–27^. While mobility data can tell us who is staying home and where people are going when they leave the home, internet volume data provides a unique perspective on what is happening within households, particularly in relation to adapting work arrangements to COVID-19 lockdown requirements.

Here, we demonstrate how relationships between occupational factors and home internet traffic can provide insight into social and economic disparities that are amplified by the pandemic and associated mitigation strategies. Our results support the hypothesis that occupational factors link the ability to work from home with income security, and clearly show how this link produces strong positive correlations between income security and increased home internet activity during COVID-19 restrictions. Our results in the Australian context may help explain observations from other recent studies describing the connection between income, internet, and the ability to self-isolate during COVID-19^17,21^. Overall, the results we present contribute to the emerging picture of the impacts of COVID-19 on human behaviour, and will help policy makers to understand the balance between public health and social impact in making future decisions. Furthermore, results such as those presented in this work will contribute to the ability to produce precise, integrated models of epidemic dynamics connected to social and economic phenomena.

## Methods

### Data sources

Our study examines the Greater Metropolitan Areas of Melbourne and Sydney, Australia. To subdivide these regions, the geographic analysis unit adopted was the ASGS Statistical Area Level 2 (SA2). SA2 regions are defined by the Australian Bureau of Statistics (ABS) and typically contain between 1000 and 10,000 residents, representing neighbourhoods that socially and economically interact^28^. Aggregating to this geographic unit, we used the following data sets to create our measures of occupational factors, income security and internet usage:

- Detailed population surveys quantifying the distributions of occupation types within local regions, and the characteristics of different occupation classifications^29–32^.
- A population-scale data set describing home internet use patterns aggregated on the scale of SA2. This data was obtained from nbn co ltd. (**nbn**), a Government Business Enterprise providing national wholesale broadband access in Australia.
- Recent survey data collected in September, 2020 by the COVID-19 Attitudes Resilience and Epidemiology (CARE) study to substantiate our individual-level interpretation of observed population-scale trends^33^.

All survey data used in this study was collected with the informed consent of all participants. The CARE study was by approved by the University of Melbourne Human Research Ethics Committee (2056694). Ethics approval applied to all study sites. All methods were carried out in accordance with relevant guidelines and regulations.

### Computation of income security by occupation

To quantify the salient features of the complex distributions of employment characteristics in Sydney and Melbourne, we constructed an income security index using data on employment security and income characteristics linked to the Australian and New Zealand Standard Classification of Occupations (ANZSCO). For income, we used average weekly earnings by occupation from the ABS Census of 2016^34^. To calculate the employment security associated with an occupation, we used the most recent iteration (2018) of the nationally-representative Household, Income and Labour Dynamics in Australia (HILDA) survey. For each occupation, we computed income security as the product of the proportion of securely employed HILDA respondents, and the average weekly wage reported by the ABS (with income rescaled to the sample maximum). This gives a value between 0 and 1, with zero corresponding to occupations with no securely employed individuals and values of 1 corresponding to occupations with maximal remuneration as well as 100% securely employed respondents. The distributions of these values and the component measures of income and proportion securely employed are shown in the Supplementary Fig. S3.

Occupation security status was developed as an individual categorical variable with two levels: [0 (secure employment)] or [1 (insecure employment)]. An individual was classified as ‘secure’ if they had a fixed-term or permanent job. We computed the employment security score associated with each occupation as the proportion of respondents in each occupation who were securely employed. We then computed the index of income security by occupation as the product of the employment security score and average weekly earnings (rescaled to the sample maximum). Distributions of the resulting income security values and the component measures of income and proportion securely employed are shown in Supplementary Fig. S3. Note that those on a fixed-term contract were classified as securely employed because the work conditions associated with fixed-term employment are more similar to the conditions of permanent employment than they are to casual work conditions. Those on fixed-term contracts have also been shown to be more sociodemographically similar to those employed permanently than to those employed on a casual basis^35,36^.

### Computation of ability to work from home by occupation

To compute the working from home indicator for each occupation classification, we adapted an analysis done by Dingel and Neiman^37^ (available on GitHub, https://github.com/jdingel/DingelNeiman-workathome) to establish which occupations could potentially be performed from home. Dingel and Neiman used the ‘Work Context’ and ‘Generalized Work Activities’ occupational surveys from the O*NET®Database. Drawing on a series of questions, they classified occupations according to whether they were compatible with working from home. For example, occupations were considered to be unsuitable for working from home if they involved activities that required a workplace such as operation of machinery or handling of specialised items (for more information in methodology, please refer to Dingel^37^). To produce international estimates we linked the binary work-from-home classifications (N = 969) produced by Dingel and Nieman, to International Standard Classification of Occupations (ISCO-08) 4-digit codes. This data (N = 1024) was then linked to the Australian and New Zealand Standard Classification of Occupations (ANZSCO) 6-digit codes and subsequently aggregated to the level of ANZSCO 4-digit codes for compatibility with ABS occupation distributions by SA2. During this linking and aggregation process, some of the occupation codes did not link (N = 36). For these, binary values were manually determined by referring to similar occupation descriptions from the original O*Net and SOC descriptions.

### Income security and ability to work from home as SA2-level measures

To quantify the ability to work from home and income security (respectively) for each SA2, we computed the weighted average of the respective variables by occupation based on the occupation distribution in each region. Weights correspond to the fraction of employed individuals working in each occupation within each SA2 region as reported by the ABS Census of 2016. The distributions of employed persons by occupation (4-digit level), by SA2 region was generated using the Census TableBuilder application provided by the ABS^30^. The data set queried was the 2016 Census Count of Persons by Usual Residence, aggregated by OCCP 4-Digit Level, by SA2 (UR). Distributions of the resulting SA2-level values and the component measures are shown in Supplementary Fig. S5.

### Analysis of internet usage data from **nbn**

**nbn** provided access to aggregated Australian internet usage volume data from household customers. The data provided by **nbn** consist of upload and download volume (in bytes) by individual households over 30 min intervals, spatially aggregated into SA2 regions. Different types of internet usage (such as streaming movies, videoconferencing and online gaming) are associated with different patterns of upload and download volume. The high time resolution and structure of the data allowed us to approximately differentiate between background (latent) internet activity, and active internet use. The data were restricted to the total download and upload volume for 30 min intervals, per SA2 region, and the corresponding number of active internet connections per time slot that generated these data. Only data generated by at least 50 domestic connections were provided, to avoid privacy concerns and to reduce the impact of aberrant individual household behaviours in regions with insufficient service coverage.

Outlier data points beyond three standard deviations from the corresponding time period mean were removed, to limit the impact of outlier usage data points, usually caused by network management or internal infrastructure configuration changes. We set the data collection interval of October 10th, 2019 until November 29th, 2019 as the baseline period, when life in Australia was not impacted by school holidays or major, longer public holidays, and preceding the major disturbance produced by the bushfire season of summer 2019–2020. The period representing behaviour during the 1st wave of COVID-19 restrictions was set to the interval of April 18th–April 24th, 2020, while the period representing the second wave of restrictions was set to the interval of August 8th–August 14th, 2020.

**Figure 1:**
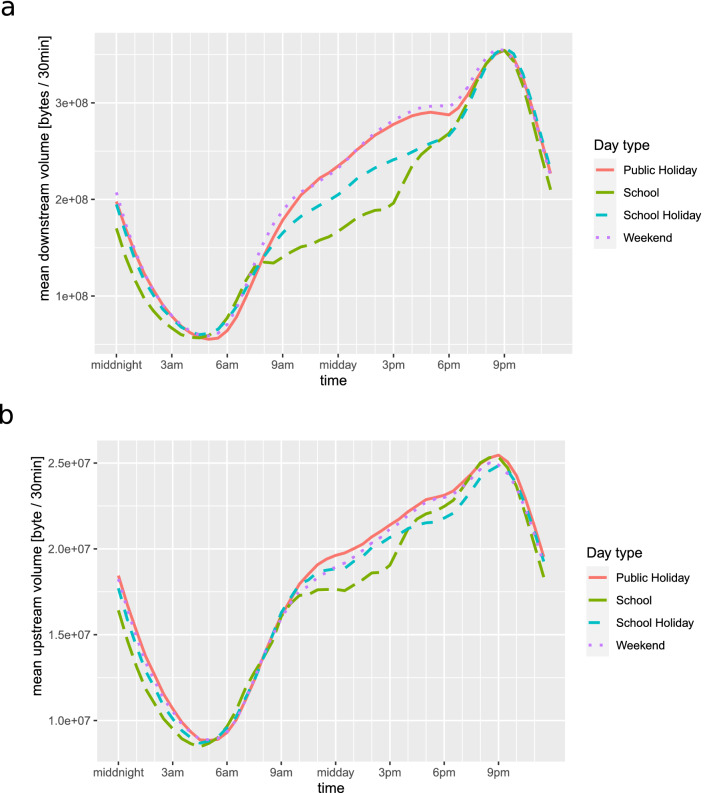
Long-term daily fluctuation of average internet activity per active service, per 30min period, for downloads (**a**), and uploads (**b**). The daily minimum and maximum, as well as the daytime plateau of activity are clearly visible. The changes between normal work days (school days), weekends and public holidays are salient. The daily trends in this figure were aggregated over the period from Sep. 1st 2019, to Sep. 1st 2020.

The following upload and download volume characteristics per SA2 region were computed for these three periods (baseline and the two COVID-19 intervals): (1) an overall daytime average volume; (2) the daily average minimum volume relating to the minimum internet usage, between 4:30 a.m. and 5:30 a.m.; (3) the average volume generated during the daytime period (from school start until noon, 9:00 a.m.–12:00 p.m. noon); and (4) the average daily maximum usage (8:30 p.m.–10:00 p.m.). Figure 1 demonstrates the daily fluctuation of average internet use for the period of September 2019–September 2020 in Australia.

### Censoring of outlier data for correlation analysis

All correlation coefficients were computed after censoring data points for which either variable was greater than three standard deviations from the sample mean. The full data set made available with this article contains all values including outlier data. Outlier data is not included in the scatter plots shown in Figs. 5 and 6.

### Analysis of data from the CARE survey

Our analysis used data from the CARE study’s Victoria-wide survey which aimed to address the overall question: How were Victorians thinking, feeling and behaving in response to the ‘second wave’ of the COVID-19 epidemic and the associated public health measures. The survey was self-administered online in English to 1006 Victorian residents aged 18 years and over. The survey was based on research developed and conducted by Imperial College in the UK in mid-March 2020^33,38^. Some questions in the Australian survey were modified slightly to reflect local response measures and terminology. Additional questions were added to the Australian survey to measure social and emotional impacts. Data collection in both the UK and Australia was conducted by the online market research agency YouGov.

The CARE study used a structured questionnaire addressing the following three domains: perceptions of risk and consequences of COVID-19 infection; measures taken by individuals to protect themselves and others from COVID-19 infection; and social and emotional impact. The questionnaire was administered online to members of the YouGov Australia panel of individuals who have agreed to take part in surveys of public opinion (over 120,000 Australian adults). Panellists, selected at random from the base sample, received an email inviting them to take part in a survey, which included a survey link. Once a panel member clicked on the link and logged in, they were directed to the survey most relevant to them available on the platform at the time, according to the sample definition and quotas based on census data. A plain language statement appeared on screen and respondents were required to electronically consent prior to the survey questions appearing. Proportional quota sampling was used to ensure that respondents were demographically representative of the Victorian adult population, with quotas based on age, gender, household income, location (state and metropolitan or regional) and whether a language other than English is spoken at home.

The study was by approved by the University of Melbourne Human Research Ethics Committee (2056694). Ethics approval applied to all study sites.

## Results

Our analysis focused on the urban areas of Sydney and Melbourne during a pre-COVID period (which we use as a baseline), during the first pandemic wave in March and April 2020 (with Australia-wide transmission and mobility restrictions), and during the second wave from July 2020 (with substantial transmission and mobility restrictions in Melbourne but not in Sydney). We identify positive correlations between income security and changes to internet activity during COVID-19. These correlations are consistent with the hypothesis that higher income security is associated with more people working from home during lockdown. This hypothesis is further supported by individual-level data from the CARE survey. We observe that in Sydney this trend persists after the release of lockdown restrictions, indicating the possibility of a ‘new normal’ of remote working conditions, particularly for occupations associated with higher income security. In Melbourne, we find that the role of children conducting their studies online disrupts these correlations due to an inverse relationship between income security and the proportion of families with children.

### Employment, income security, and the ability to work from home

**Figure 2:**
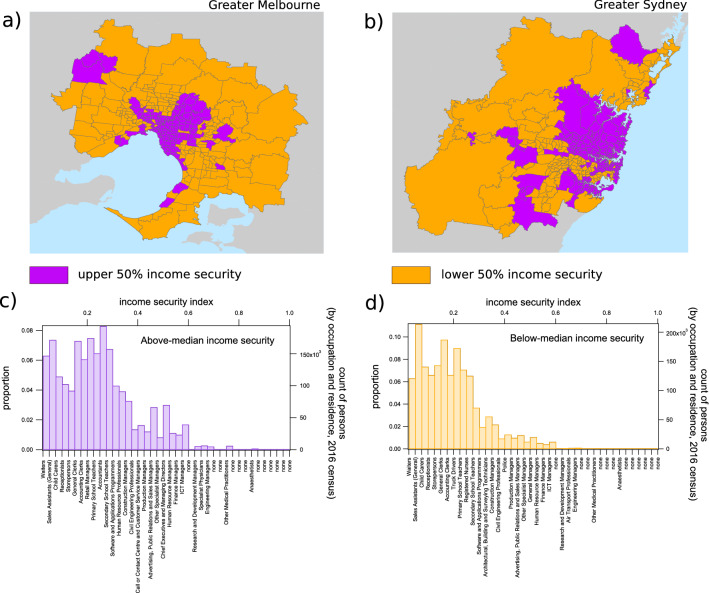
Geospatial and occupational distribution of income security. The choropleth maps in (**a**) and (**b**) demonstrate the geospatial distribution of income security in Greater Melbourne and Greater Sydney (respectively) on the scale of Statistical Area Level 2 (SA2). Areas below the median income security index of 0.1809 are colored orange while those above the median are colored purple. The histograms in (**c**) and (**d**) demonstrate the distribution of the population in these regions over the income security spectrum, with indicative occupation types for each bin shown in the bottom y-axis labels (the label corresponding to the most prevalent occupation classification for each bin is shown). For both histograms, waiters have the lowest income security, while anaesthetists have the highest.

Income security is distributed spatially according to distinct patterns, with high values in the central and northeast suburbs of both Sydney and Melbourne (Fig. 2a,b). The upper 50% income security quantile (Fig. 2c) favours managerial and office-based occupations, while the lower 50% quantile (Fig. 2d) contains more service staff and other socially-oriented occupations. The frequency distributions of average income security among SA2s in Sydney and Melbourne (respectively) are provided in the Supplementary Fig. S4, which demonstrates that the distributions in the two regions are not significantly different (two sample t-test, $p = 0.552$). High resolution choropleth maps of income security by region can be found in the Supplementary Fig. S6a.

**Figure 3:**
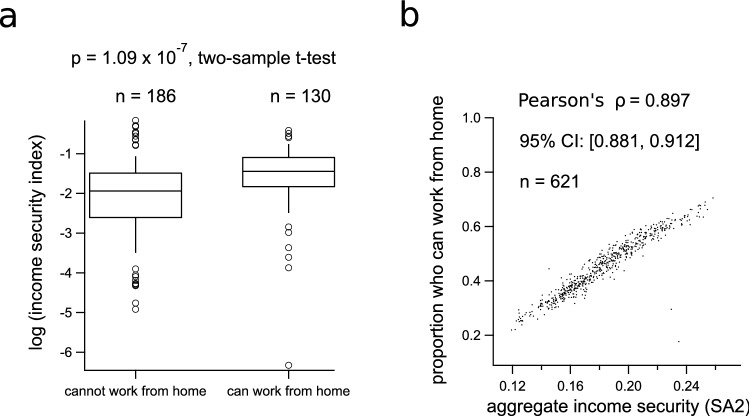
Relationship between income security and the ability to work from home. Box plots in (**a**) compare the distributions of log-transformed income security, grouped by the ability to work from home for each of 321 occupations classified by 4-digit ANZSCO codes. The distributions in (**a**) are computed from the HILDA survey, 2018. The scatter plot in (**b**) demonstrates the correlation between average income security for each SA2 region, and the corresponding average proportion of individuals who can work from home, computed from the occupation distribution of each SA2 in the Greater Sydney and Greater Melbourne regions released in the 2016 ABS Census. [Note: the log-transformed income security data in (**a**) omits 24 occupations that had a value of 0 for income security (no securely employed HILDA respondents), of these, 2 were grouped into the “can work from home” category and 22 into the “cannot work from home” category].

To examine the qualitative association between income security and the ability to work from home indicated by the distributions in Fig. 2c,d, we apply the occupation classification method developed by Dingel and Neiman^37^. This results in a binary (0 or 1) value indicating whether or not a particular occupation type can be performed from home. We found a strong association between income security and the ability to work from home (Fig. 3). This association was observed both by occupation (Fig. 3a) and by geographic region (Fig. 3b). See the Supplementary Fig. S5 for histograms of the distributions shown in Fig. 3a as well as the distributions of the x- and y-axis variables used in Fig. 3b.

### Changes to internet traffic during COVID-19

**Figure 4:**
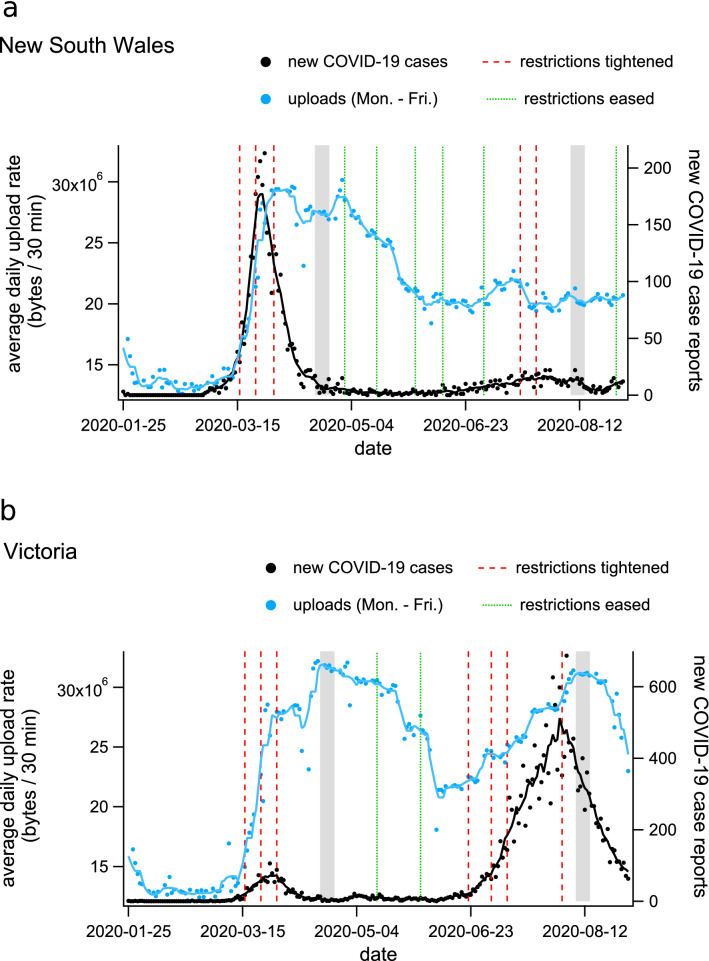
Timeseries plots of average daytime internet use, COVID-19 case incidence, and restriction policy implementation for (**a**) New South Wales and (**b**) Victoria, Australia. Daily average upload rates per household, per 30 min interval between 9 a.m. and 12 p.m. are shown as blue dots for weekdays (blue dots, the blue line is the 7-day average). Daily case incidence is shown as black dots (the black line is the 7 day average), and dates on which restriction policies were modified are shown as vertical dashed lines for increasing (red) and decreasing (green) restriction levels. The grey bands indicate the dates over which **nbn** data was averaged for our analysis of first- and second-wave changes. See the Supplementary Fig. S1 for an equivalent timeseries presenting average downloads rather than uploads.

To quantify changes to home internet use during COVID-19 restrictions, we aggregated internet activity data from all SA2 regions within Greater Sydney and Greater Melbourne (respectively). Over the pre-COVID baseline, we averaged the per-user upload and download rates from the hours of 9 a.m. to 12 p.m. in order to capture a baseline measurement of putative remote work-related internet activity (see Methods). During the first and second waves of COVID-19 in Australia, peaks in case incidence coincided with the implementation of the most restrictive policies, and were followed by increases in total internet use, which peaked approximately 1 to 3 weeks after implementation of the tightest level of restrictions (Fig. 4).

After identifying time intervals representative of the changes induced by the first- and second-waves of restrictions, we examined spatial variation among individual SA2 regions during those periods. The grey bands in Fig. 4 show the periods over which **nbn** data was averaged for each individual SA2 in order to examine the spatial distribution of changes to internet activity during first and second waves of COVID-19. For visualisation of spatial trends, high-resolution choropleth maps of internet activity changes relative to baseline can be found in the Supplementary Fig. S6b,c.

**Figure 5:**
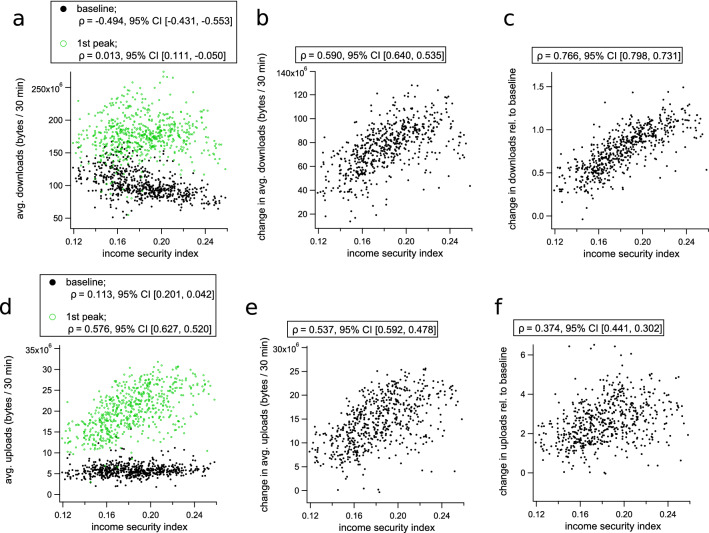
Changes to internet use during the 1st wave of COVID-19 restrictions in Australia, plotted against income security for each SA2 region. (**a**) Shows absolute average household download rates before (black dots) and during (green dots) the selected period (April 18th to April 24th, 2020). (**b**) Plots the absolute change in average per-household download rate during the first-wave period, and (**c**) plots the change in download rate relative to baseline. (**d**) Shows absolute upload rates before (black) and during (green) COVID-19, while (**e**) shows absolute changes to upload volume, and (**f**) shows changes to upload volume relative to baseline. In each subplot, the internet traffic quantifiers are plotted against the income security score for the corresponding SA2 region, and Pearson’s correlation coefficients with 95% CI intervals are shown in the legends.

We found that during the first period of restrictions, areas with higher income security tended to exhibit larger increases in internet volume per household (Fig. 5a–c). However, these trends were produced by qualitatively different changes for downloads and uploads, respectively:

- During the pre-COVID baseline period, absolute download volume tended to decrease with income security, becoming uncorrelated during the first wave of restrictions (Fig. 5a). This transition produces larger increases in download volume in areas with higher average income security (Fig. 5b,c).
- On the other hand, absolute upload volume shows baseline rates that are initially uncorrelated with income security and transition to an increasing trend during the first wave of restrictions (Fig. 5d). This produces changes in upload volume that have similar correlations with income security to those observed for downloads (Fig. 5e,f), but that occur due to the emergence of a positive correlation rather than the removal of a negative correlation with the onset of restrictions.

The negative baseline trend of download rates with income security may result from the activity of children. We observed a strong positive correlation between the proportion of families with children and baseline download rates ($\rho ~=~0.72~95\%\;\text {CI}~[0.68,~0.75]$), and a negative correlation between the proportion of families with children and income security ($\rho ~=~-0.39~95\%\;\text {CI}~[-0.45,~-0.32]$). This suggests that children engaged in online activity may establish the negative baseline correlation between download rates and income security (Fig. 5a).

Children’s activities also appear to influence the changes observed during lockdown. During the time interval selected to represent the first wave of restrictions (April 18th to April 24th), school holidays were still in effect in Greater Sydney while in Melbourne, children had returned to their studies remotely. Because regions with higher income security tend to have a lower proportion of families with children, remote learning activity weakens the positive association between upload activity and income security produced by adults working from home. Correlations between income security, the proportion of families with children, and internet activity in Sydney and Melbourne (respectively) during the first wave of COVID-19 restrictions are shown in the Supplementary Tables S2 and S3.

For the second wave of COVID-19 (and associated restrictions), we selected the appropriate time period using internet data from Victoria, where the second epidemic wave was concentrated. In Victoria during the second wave, internet activity peaked during the week of August 8th to August 14th. As for the first wave, this home internet activity peak immediately followed the implementation of the highest level of restrictions (Fig. 4b).

Because of the substantially different epidemiological and policy situations in Sydney (New South Wales) and Melbourne (Victoria) during the second-wave period (Fig. 4), we examined the relationship between internet traffic, lockdown policy, and income security for each city separately. Comparing the two cities provides insight regarding changes in behaviour related to the contrasting scenarios. During the second wave, Greater Sydney experienced a series of localised outbreaks with minimal social restrictions, while in Melbourne there was a large-scale epidemic with mandatory movement restrictions.

**Figure 6:**
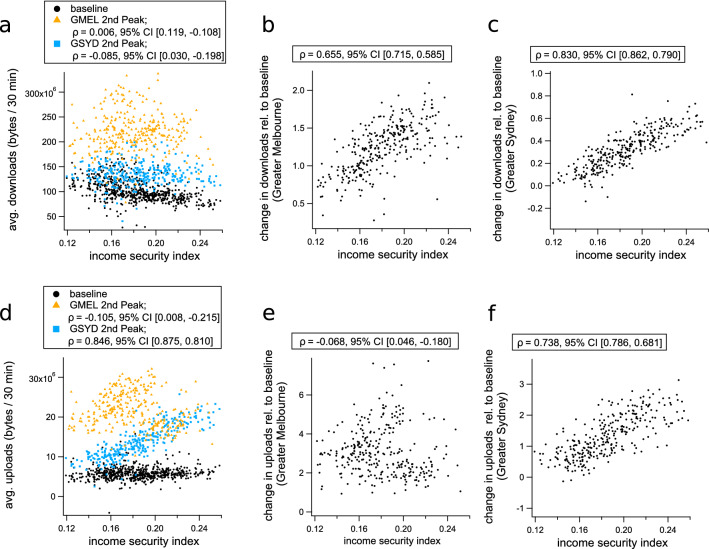
Changes to internet use during the second wave of COVID-19 restrictions in Australia, plotted against income security for each SA2 region. (**a**) Shows absolute download volumes averaged over the baseline period and second wave of COVID-19 restriction policies (August 8th to August 14th, 2020), which includes baseline (black dots), 2nd wave values for Greater Sydney (blue squares), and second wave values for Greater Melbourne (orange triangles). Plots (**b**) and (**c**) show the change in download volumes relative to pre-COVID baseline as a function of income security index for each SA2 in Sydney and Melbourne, respectively. Plots (**d**), (**e**) and (**f**) show the same analysis as (**a**), (**b**), and (**c**), respectively, for uploads rather than downloads. In each subplot, the internet traffic quantifiers are plotted against the income security score for the corresponding SA2 region, and Pearson’s correlation coefficients with 95% CI intervals are shown in the legends.

While household internet traffic declines in Sydney during the second wave relative to the first wave, the positive correlation between income security and internet activity relative to baseline remains prominent for both downloads (Fig. 6a,c), and uploads (Fig. 6d,f). This is despite the absence of formal stay-at-home orders in the Greater Sydney region at that time (though some restrictions on social gatherings remained in place). The time interval between the first and second waves was long enough to support the assertion that behavioural changes made in response to COVID-19 lockdown policies remain observable after those policies have been formally relaxed.

Greater Melbourne behaves similarly in both waves with respect to changes in download traffic as a function of income security (compare Figs. 5a,c, 6a,b). However, changes to upload volumes do not mirror the correlations observed during the first wave (compare Figs. 5d,f, 6d,e). In fact, there are many areas of Melbourne with high income security that show substantial reductions in upload traffic during the second wave, relative to the first. While our data gives no immediate explanation for this counter-intuitive trend, we speculate that it may be due to alterations in work habits that occurred as the lockdown became protracted. Decreases in upload traffic without corresponding decreases in download traffic could result from individuals continuing to perform work activities from home, but participating in less “face-to-face” online interaction. Conversely, widespread adoption of remote schooling practices could help explain the increase in upload rates for regions in the mid-range of the income security spectrum. Such an effect is consistent with the weak but positive correlations between the proportion of families with children and changes to upload volumes ($\rho = 0.15~ 95\%~ \text {CI}~ [0.033, 0.25]$, see Supplementary Table S5). This suggestion is also consistent with the observation that daytime internet activity increases during school holidays, when children are more likely to be in the home (Fig. 1b).

We hypothesise that schooling in the home had a greater impact on internet volume in general, and upload rates in particular, than working remotely from home during the second wave of COVID-19 restrictions in Melbourne. This hypothesis is supported by a preliminary principal component analysis, summarised in the Supplementary Fig. S2. This 3-component PCA shows an increased role of children in determining upload rates in Greater Melbourne during the second-wave period. Specifically, Supplementary Fig. S2 demonstrates a qualitative change in the relationship between the proportion of families with children, income security, and second-wave changes to upload activity. Upload rate is positively associated with the proportion of families with children in the first component (explaining 55% of the variance) and positively associated with income security in the second component (explaining 32% of the variance). In both components, income security and the proportion of families with children are negatively associated. These PCA results support the suggestion that occupation-related correlations between net changes to upload activity and income security are disrupted by the activities of children in online schooling. This may be explained as a competing effect because lower average income security is associated with a higher proportion of families with children ($\rho = -0.45~ 95\%~ \text {CI}~ [-0.53, -0.35]$, Supplementary Table S5), while the capacity to work from home increases with income security ($\rho = 0.90~ 95\% ~\text {CI}~ [0.88, 0.91]$, Fig. 3b).

### Change of work environment for above- and below-median income earners

To confirm that the household-level trends inferred from SA2-level aggregate variables corresponded to observations made on the individual level, we analysed representative data from Victoria collected by the CARE study. While the CARE study did not collect data on income security per-se, it did record the annual income bracket reported by each respondent.

One of the survey questions was posed as follows: ‘Have you personally experienced a change in work environment (working from home) because of COVID-19 and the measures to prevent its spread? (yes or no)’. We computed the proportion of respondents who reported income above and below (or within) the median income bracket for the sample (sample median annual income was $AUD 60,000 to 69,999) who answered ‘yes’ to this question. We then performed a two-tailed Fisher’s exact test to determine the resulting odds ratio between the two groups, and its statistical significance given the response numbers (see Table 1). The results demonstrate a strong positive relationship between income and switching to work from home, with an odds ratio of 2.15 (95% CI [1.59, 2.92], $$p = 6.8\times 10^{-7}$$) computed for the above-median income group, relative to the median-and-below income group. While the income data tabulated by the CARE survey is not an exact representation of the income security score used in our analysis of internet trends (which incorporated contract classification), this result supports the same conclusion: those with higher financial security have more capacity to change their work environments in response to COVID-19 restrictions.

**Table 1:** CARE survey results for income and working from home due to COVID-19 (Victoria).

Changed work environment?	Yes	No	Total
Above median income	144	267	411
Below median income	93	371	464
Total	237	638	875

From the CARE survey: comparison between above and below-median income groups’ responses to the question: ‘Have you personally experienced a change in work environment (working from home) because of COVID-19 and the measures to prevent its spread? (yes or no)’.

In summary, our results support the hypothesis that occupational factors link the ability to work from home with income security, and clearly show how this link produces strong positive correlations between income security and increases to home internet activity during COVID-19 restrictions. These correlations are consistent with the assertion that higher income security is associated with more people working from home during lockdown. This assertion is further supported by individual-level data from the CARE survey. We observe that in Sydney this trend persists after the release of lockdown restrictions, indicating the possibility of a ‘new normal’ of remote working conditions, particularly for occupations associated with higher income security. In Melbourne, we find that the role of children conducting their studies online disrupts these correlations due to an inverse relationship between income security and the proportion of families with children.

## Discussion

By combining an analysis of occupational factors and distributions with large-scale, high-resolution, real-time data on internet activity, we have broadly characterised the impact of COVID-19 restrictions on two major urban centres in Australia, demonstrating three main findings. First, that occupations associated with greater income security are also associated with the ability to work from home. Second, that Internet usage increased during periods in which COVID-19 restrictions were in place. Increases were greatest in regions with high income security, suggesting that they may be caused by people who were able to adapt to working from home. Finally, that during the second wave in Melbourne, lower-income regions also displayed increased internet usage, likely driven by increased levels of remote schooling.

These findings confirm and elaborate on the general observation that COVID-19 and the associated restrictions on human activity distort normal life activities, with the relative impact largely determined by occupational and demographic factors^39–42^. This unequal impact is dominated by two major influences: the types of occupations in which people are engaged, and the compositions of households and families. Our analysis helps to illustrate how life changed during lockdown. For households with no children, and members engaged in work that could be conducted from home, the internet provided a means of continuing livelihood during the prolonged periods of mobility restrictions implemented to combat virus transmission. Furthermore, the members of such housholds were likely to have been previously employed in high-income occupations with relatively strong employment guarantees, adding a measure of confidence in the ability to financially out-last the economic downturn. On the other hand, households with lower income security were also less likely to have been able to work from home, and were more likely to have had children who required care during school closures. For such households, life during lockdown was financially insecure, potentially stressful on the family, and came with a heightened risk of exposure to the pandemic virus due to the work requirements of those occupations which remained active.

### Study limitations

Due to the nature of the data we analysed, our study has several limitations. With the exception of the CARE survey results, all of the data analysed in this work is aggregated to sub-populations. Therefore, a direct behavioural interpretation of the correlations we report is contingent on the assumption that the variables we investigate are independently distributed within these sub-populations. While there are likely to be exceptions, the spatial aggregation of areas by income security (Fig. 2a,b), suggests that the spatial resolution of SA2 regions is sufficient to sample within the boundaries that define salient heterogeneity of the population for the purposes of our study. To confirm this quantitatively, the spatial autocorrelation (Moran’s I) between neighbouring SA2 regions is shown in the Supplementary Sect. S6. This analysis shows highly significant spatial autocorrelation of all relevant variables (income security, and changes to internet usage volumes). From this we conclude that the SA2 scale is an appropriate resolution for the spatially varying quantities studied in this work.

Another inherent limitation is introduced though the use of household internet data in quantifying behaviour across the income spectrum: home internet connections have financial requirements including usage fees and installation costs that may be prohibitive for those at the low end of the income spectrum. For example, a study in the United States recently determined that household internet speeds typically increase with income, and the combination of both high income and high-speed internet is associated with an enhanced ability to self-isolate during the pandemic^21^. Our analysis of relative changes in data volume per active connection addresses potential inequity in the spatial distribution of broadband infrastructure as well as potential correlation of available bandwidth and income security. However, it does not address the possibility that lower-income households use access plans with smaller usage caps. Such a trend could complicate the interpretation of changes to internet volume with respect to working from home during lockdown. The analysis presented here assumes that larger increases to internet usage are indicative of a higher proportion of resident individuals switching to home-based work. An alternative interpretation of the observed trends could be that the proportion of individuals working from home does not depend on income security, but that households with higher income security have access plans with higher usage caps. This alternative interpretation requires data caps to regularly limit internet usage. While such a scenario is possible, we believe it to be less plausible than the one we chose to assume. In addition, the individual-level CARE survey analysis supports our chosen interpretation.

A related limitation is that households with no internet connection were implicitly excluded from our analysis. Using Australian data from the 2016 ABS Census, we computed a correlation of $\rho = 0.50,~95\%~\text {CI}~[0.44,~0.56]$, associating the fraction of households with an internet connection (as of 2016) with the income security measure computed here (see Supplementary Table S1). Therefore, our use of home internet traffic data to estimate the ability of workers with differing income security to adapt to COVID-19 restrictions may omit the behaviour of many low-income households, producing an underestimate of the effects of occupational factors on behaviour during the crisis.

## Supplementary Information

Supplementary Information.

## Data Availability

All processed data necessary for reproducing the figures and results of this paper is available in the GitHub repository located here: https://github.com/cjzachreson/Internet_Income_and_COVID19_in_Australia. Access to raw data from the HILDA survey, **nbn**, and CARE survey was obtained under restricted access agreements and cannot be made directly available. However, arrangements for access may be made for eligible researchers. All ABS data can be accessed by following the instructions provided here: https://www.abs.gov.au/websitedbs/D3310114.nsf/home/How+to+Apply+for+Microdata.

## References

[1] International Labour Organisation (ILO). COVID-19 and the World of Work: Country Policy Responses. ILO, Geneva, Switzerland. (2020). [online]. https://www.ilo.org/global/topics/coronavirus/regional-country/country-responses/lang--en/index.htm. Accessed 10 Nov 2020.

[2] Australian Government. *The Treasury* (Three-month review, The JobKeeper Payment, 2020). [online]. https://treasury.gov.au/publication/jobkeeper-review. Accessed 16 Oct 2020.

[3] Phillips B, Gray M, Biddle N (2020). COVID-19 JobKeeper and JobSeeker Impacts on Poverty and Housing Stress Under Current and Alternative Economic and Policy Scenarios.

[4] Anderson G, Frank JW, Naylor CD, Wodchis W, Feng P (2020). Using socioeconomics to counter health disparities arising from the COVID-19 pandemic. BMJ..

[5] Bambra C, Riordan R, Ford J, Matthews F (2020). The COVID-19 pandemic and health inequalities. J. Epidemiol. Community Health.

[6] Cubrich M (2020). On the frontlines: Protecting low-wage workers during COVID-19. Psychol. Trauma Theory Res. Pract. Policy.

[7] Fisher J, Languilaire JC, Lawthom R, Nieuwenhuis R, Petts RJ, Runswick-Cole K (2020). Community, work, and family in times of COVID-19. Community Work Fam..

[8] Sanderson K, Cocker F (2013). Presenteeism: Implications and Health Risks. Aust. Fam. Physician.

[9] Abedi, V., Olulana, O., Avula, V., Chaudhary, D., Khan, A., Shahjouei, S. *et al*. Racial, economic, and health inequality and COVID-19 infection in the United States. *J. Racial Ethn. Health Disparities* **8**(3), 1–11 (2020). 10.1007/s40615-020-00833-4 PMC7462354 32875535

[10] Lee, K., Sahai, H., Baylis, P., Greenstone, M. Job Loss and Behavioral Change: The Unprecedented Effects of the India Lockdown in Delhi. University of Chicago, Becker Friedman Institute for Economics Working Paper. (2020-65) (2020).

[11] Forsythe E, Kahn LB, Lange F, Wiczer D (2020). Labor demand in the time of COVID-19: Evidence from vacancy postings and UI claims. J. Public Econ..

[12] Cajner, T., Crane, L. D., Decker, R. A., Grigsby, J., Hamins-Puertolas, A., Hurst, E. *et al*. The US Labor Market During the Beginning of the Pandemic Recession. (National Bureau of Economic Research, 2020). [online]. https://www.nber.org/papers/w27159. Accessed 21 Nov 2020.

[13] Adams-Prassl A, Boneva T, Golin M, Rauh C (2020). Inequality in the impact of the coronavirus shock: Evidence from real time surveys. J. Public Econ..

[14] Couch KA, Fairlie RW, Xu H (2020). The impacts of COVID-19 on minority unemployment: First evidence from April 2020 CPS microdata. Available SSRN.

[15] Montenovo, L., Jiang, X., Rojas, F. L., Schmutte, I. M., Simon, K. I., Weinberg, B. A. *et al*. Determinants of Disparities in COVID-19 Job Losses. (National Bureau of Economic Research, 2020). [online]. https://www.nber.org/papers/w27132. Accessed 19 Nov 2020.

[16] Brough, R., Freedman, M., & Phillips, D. Understanding Socioeconomic Disparities in Travel Behavior During the COVID-19 Pandemic. University of California, Irvine Department of Economics Working Paper Series. (2020). 10.1111/jors.12527 PMC8251298 34230690

[17] Jay J, Bor J, Nsoesie EO, Lipson SK, Jones DK, Galea S (2020). Neighbourhood income and physical distancing during the COVID-19 pandemic in the United States. Nat. Hum. Behav..

[18] Brynjolfsson, E., Horton, J. J., Ozimek, A., Rock, D., Sharma, G., TuYe, H. Y. COVID-19 and Remote Work: An Early Look at US Data. (National Bureau of Economic Research, 2020). [online]. https://www.nber.org/papers/w27344. Accessed 22 Nov 2020.

[19] Bonaccorsi G, Pierri F, Cinelli M, Flori A, Galeazzi A, Porcelli F (2020). Economic and social consequences of human mobility restrictions under COVID-19. Proc. Natl. Acad. Sci..

[20] Weill JA, Stigler M, Deschenes O, Springborn MR (2020). Social distancing responses to COVID-19 emergency declarations strongly differentiated by income. Proc. Natl. Acad. Sci..

[21] Chiou, L. & Tucker, C. Social Distancing, Internet Access and Inequality. (National Bureau of Economic Research, 2020). [online]. https://www.nber.org/papers/w26982. Accessed 22 Nov 2020.

[22] Feldmann, A., Gasser, O., Lichtblau, F., Pujol, E., Poese, I., Dietzel, C. *et al*. The lockdown effect: Implications of the COVID-19 pandemic on internet traffic. In *Proceedings of the ACM Internet Measurement Conference* 1–18 (2020).

[23] Zachreson, C., Mitchell, L., Lydeamore, M., Rebuli, N., Tomko, M., & Geard, N. Risk mapping for COVID-19 outbreaks using mobility data. arXiv preprint arXiv:2008.06193. (2020). 10.1098/rsif.2020.0657 PMC7879754 33404371

[24] Buckee CO, Balsari S, Chan J, Crosas M, Dominici F, Gasser U (2020). Aggregated mobility data could help fight COVID-19. Science (New York, NY)..

[25] Pepe E, Bajardi P, Gauvin L, Privitera F, Lake B, Cattuto C (2020). COVID-19 outbreak response, a dataset to assess mobility changes in Italy following national lockdown. Sci. Data..

[26] Warren, M. S., & Skillman, S. W. Mobility changes in response to COVID-19. arXiv preprint arXiv:2003.14228. (2020).

[27] Chang, S. *et al.* Mobility network models of COVID-19 explain inequities and inform reopening. *Nature ***589**, 82–87 (2021). 10.1038/s41586-020-2923-3 33171481

[28] Standard, Australian Statistical Geography. & (ASGS). [online (2020) accessed 01 Jun 2021]. https://www.abs.gov.au/websitedbs/D3310114.nsf/home/Australian+Statistical+Geography+Standard+(ASGS).

[29] Survey, H. I. L. D. A. [online (2020)]. https://melbourneinstitute.unimelb.edu.au/hilda. Accessed 15 Dec 2020.

[30] TableBuilder, About. [online (2020)]. https://www.abs.gov.au/websitedbs/d3310114.nsf/home/about+tablebuilder. Accessed 12 Aug 2020.

[31] Australian Bureau of Statistics Census; [online (2016) accessed 15 Dec 2020]. https://www.abs.gov.au/census.

[32] Sila, U. & Dugain, V. Income, wealth and earnings inequality in Australia: Evidence from the HILDA Survey. OECD; 2019. [online]. https://www.oecd-ilibrary.org/economics/income-wealth-and-earnings-inequality-in-australia_cab6789d-en. Accessed 10 Nov 2020.

[33] Shearer, F. M., Meagher, N., Chavez, K. M., Carpenter, L., Pirrone, A., Quinn, P. *et al*. Promoting resilience while mitigating disease transmission: An Australian COVID-19 study. In *COVID-19 Pandemic, Geospatial Information, and Community Resilience* 347–362. (CRC Press, 2021).

[34] ABS, 2019, cat. no. 6306.0—Microdata: Employee Earnings and Hours, Australia; 2019. [online]. https://www.abs.gov.au/statistics/labour/earnings-and-work-hours/employee-earnings-and-hours-australia/latest-release. Accessed 01 Sep 2020.

[35] LaMontagne AD, Smith PM, Louie AM, Quinlan M, Ostry AS, Shoveller J (2012). Psychosocial and other working conditions: Variation by employment arrangement in a sample of working Australians. Am. J. Ind. Med..

[36] Louie A, Ostry A, Quinlan M, Keegel T, Shoveller J, LaMontagne A (2006). Empirical study of employment arrangements and precariousness in Australia. Relat. Ind. Ind. Relat..

[37] Dingel JI, Neiman B (2020). How many jobs can be done at home?. J. Public Econ..

[38] Atchison CJ, Bowman L, Vrinten C, Redd R, Pristera P, Eaton JW (2021). Perceptions and behavioural responses of the general public during the COVID-19 pandemic: A cross-sectional survey of UK Adults. BMJ Open.

[39] Karaye IM, Horney JA (2020). The impact of social vulnerability on COVID-19 in the US: An analysis of spatially varying relationships. Am. J. Prev. Med..

[40] Hankivsky, O., & Kapilashrami, A. Beyond Sex and Gender Analysis: An Intersectional View of the COVID-19 Pandemic Outbreak and Response. (Gender and Women’s Health Unit, Centre for Health Equity, Melbourne School of Population and Health Equity, 2020). [online]. https://mspgh.unimelb.edu.au/__data/assets/pdf_file/0011/3334889/Policy-brief_v3.pdf. Accessed 24 Nov 2020.

[41] Chung, H., Seo, H., Forbes, S., & Birkett, H. Working from Home During the COVID-19 Lockdown: Changing Preferences and the Future of Work. (Kent Academic Repository, University of Kent, 2020). [online]. https://kar.kent.ac.uk/83896/1/Working_from_home_COVID-19_lockdown.pdf. Accessed 24 Nov 2020.

[42] de Haas M, Faber R, Hamersma M (2020). How COVID-19 and the Dutch ‘intelligent lockdown’ change activities, work and travel behaviour: Evidence from longitudinal data in the Netherlands. Transp. Res. Interdiscip. Perspect..

