# Supplementary material for: Mapping home internet activity during COVID-19 lockdown to identify occupation related inequalities
Source: Sci Rep. 2021 Oct 26;11:21054. doi: 10.1038/s41598-021-00553-7 (PMC8548542; doi:10.1038/s41598-021-00553-7)
Supplement: Supplementary file 1 — Supplementary Information. [file 41598_2021_553_MOESM1_ESM.pdf]

Supporting Information For:
Mapping home internet activity during COVID-19 lockdown to identify occupation related inequalities

Cameron Zachreson,[1,*] Erika Martino,[2] Martin Tomko,[3]
Freya M. Shearer,[2] Rebecca Bentley,[2] and Nicholas Geard[1,4]

[1]*School of Computing and Information Systems, The University of Melbourne, Australia*
[2]*Melbourne School of Population and Global Health, The University of Melbourne, Australia*
[3]*Melbourne School of Engineering, The University of Melbourne, Australia*
[4]*Department of Infectious Diseases, Melbourne Medical School, The University of Melbourne, Australia*

(Dated: September 10, 2021)

* cameron.zachreson@unimelb.edu.au

S1. TIMESERIES OF CASE INCIDENCE AND DOWNLOAD RATES

a)

New South Wales

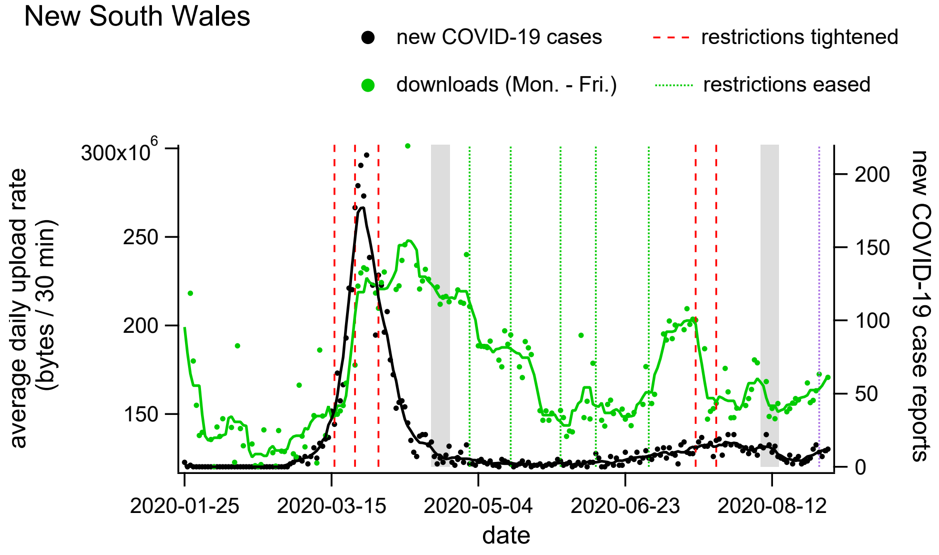

b)

Victoria

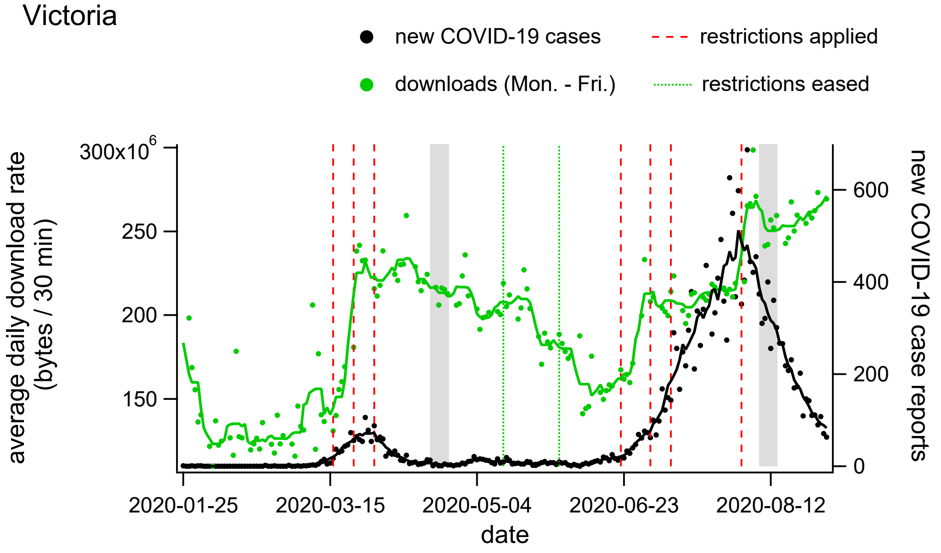

FIG. S1. Timeseries plots of average daytime internet use, COVID-19 case incidence, and restriction policy implementation for (a) New South Wales and (b) Victoria. Daily average download rates per household, per 30 min interval between 9am and 12pm are shown as blue dots for weekdays (green dots, the green line is the 7-day average). Daily case incidence is shown as black dots (the black line is the 7 day average), and dates on which restriction policies were modified are shown as vertical dashed lines for increasing (red) and decreasing (green) restriction levels. The grey bands indicate the dates over which **nbn**^{TM} data was averaged for our analysis of 1st- and 2nd-wave changes.

S2. CORRELATIONS WITH OTHER DEMOGRAPHIC FACTORS

Tables S1 through S5 show the correlation (Pearson's ρ, $\pm 95\%$ CI bounds) between various measures of internet traffic and several alternate demographic factors that we considered in our study in addition to income security.

These are:

- $p_{WFH}(MTWP)$, the proportion of individuals from each region who reported working from home in the 2016 Australian Census counts of Method of Travel to Work (MTWP). We selected this measure because it serves as an alternate measurement of the tendency to work from home under normal circumstances and could plausibly explain baseline internet traffic.

- $p_{internet}$, the proportion of households with an internet connection, as counted by the 2016 Australian Census. We chose this measure because it provides an alternate indicator of the importance of internet in normal activities.

- $p_{children}$, the proportion of families with children, as determined by the 2016 Australian Census. We chose this measure for two reasons. Firstly, because baseline internet traffic could plausibly depend on the size of a family and the presence of children and secondly, because it may help elucidate the effects of school closures on COVID-19 usage levels.

Tables S1 through S5 demonstrate the correlations of internet traffic measures with each of these factors, as well as the correlations between the demographic factors. Table S1 shows correlations between factors and internet usage for SA2 regions in Greater Sydney and Greater Melbourne during the first wave of COVID-19 restrictions. Notably, the $p_{children}$ factor appears to explain the negative correlation between baseline downloads and income security. Income security is negatively correlated with the proportion of families with children, while baseline download traffic is positively correlated. This observation indicates that the presence of children may increase internet use under normal circumstances.

The case is different for baseline upload traffic, where correlation is negligible with both income security and $p_{children}$. This distinction between upload and download traffic in the baseline data suggests that typical internet activities undertaken by children do not involve significant amounts of out-bound streaming, or two-way communication. Table S1 also shows that while download traffic during the first wave of COVID-19 restrictions correlates with the proportion of children, the change in download traffic relative to baseline is negatively

correlated. On the other hand, changes in download traffic relative to baseline correlate strongly with income security indicating that occupational factors associated with working from home dominate over the contributions of children with respect to relative changes in download activity due to COVID-19 lockdown measures. This contrast is even more apparent when examining upload traffic, for which factors associated with working from home dominate all measures of COVID-19 related changes in traffic.

Tables S2 and S3 show these correlations for Greater Sydney and Greater Melbourne, respectively, and indicate that while the two regions have qualitatively similar correlations between changes in upload volume and income security, Melbourne demonstrates a much less pronounced correspondence. We speculate that this is due to the activities of school children. During this period, students in Greater Sydney were not attending class due to the mid-semester break, while in Melbourne school was in session but schools buildings were closed and most classes were conducted remotely. The negative correlation between income security and the proportion of families with children suggests that increases in home-learning activity could disrupt occupation-related correlations between income security and increased internet traffic.

Tables S4 and S5 show correlations between demographic factors and internet traffic in the Greater Sydney and Greater Melbourne regions, respectively, during the 2nd wave of COVID-19 restrictions. During this wave, Greater Sydney did not have large numbers of cases and did not go through a second wave of mandated social distancing measures. Importantly, almost all schools were open during this period, with a few exceptions where localised outbreaks were detected in students. Comparison of correlations between internet use, income security, and $p_{children}$ indicate that work activities were the primary driver of above-baseline internet traffic during this period. The difference is particularly stark when examining upload behaviour, which is strongly positively correlated to income security and weakly negatively correlated to $p_{children}$, which suggests that deviations from baseline uploads may be a strong indicator for work-from-home behaviour, at least when schools are open.

On the other hand, Greater Melbourne shows unique correlations during the 2nd wave of COVID-19 restrictions. In Melbourne, classes in primary and secondary education were held remotely during the 1st and 2nd waves of COVID-19 restrictions, and positive correlation between income security and increased upload rates is not observed. In this case, the activity of children appears to dominate changes to internet traffic, nullifying the occupation-related correlation between income security and changes to upload rates.

Corrleation (Pearson's ρ) of internet usage with demographic variables
(Greater Sydney and Greater Melbourne, COVID-19 1st wave)

	income security	$p_{WFH}(MTWP)$	$p_{internet}$	$p_{children}$
ds_{baseline}	-0.49 [-0.55, -0.43]	-0.51 [-0.57, -0.45]	0.062 [-0.019, 0.14]	0.72 [0.68, 0.75]
ds_{peak1}	0.031 [-0.05, 0.11]	-0.088 [-0.17, -0.0079]	0.52 [0.46, 0.58]	0.69 [0.64, 0.73]
Δds_{peak1}	0.59 [0.53, 0.64]	0.45 [0.38, 0.51]	0.71 [0.66, 0.75]	0.2 [0.12, 0.27]
$\Delta ds_{\text{peak1}}/ds_{\text{baseline}}$	0.77 [0.73, 0.8]	0.66 [0.62, 0.71]	0.49 [0.42, 0.55]	-0.27 [-0.34, -0.19]
us_{baseline}	0.12 [0.042, 0.2]	0.082 [0.0011, 0.16]	0.19 [0.11, 0.26]	0.13 [0.048, 0.21]
us_{peak1}	0.58 [0.52, 0.63]	0.38 [0.31, 0.45]	0.49 [0.42, 0.55]	-0.082 [-0.16, -0.0013]
Δus_{peak1}	0.54 [0.48, 0.59]	0.39 [0.32, 0.45]	0.44 [0.37, 0.5]	-0.11 [-0.19, -0.035]
$\Delta us_{\text{peak1}}/us_{\text{baseline}}$	0.37 [0.3, 0.44]	0.26 [0.19, 0.34]	0.25 [0.17, 0.32]	-0.17 [-0.25, -0.094]
income security	1	0.7 [0.65, 0.74]	0.5 [0.44, 0.56]	-0.39 [-0.45, -0.32]
$p_{WFH}(MTWP)$	0.7 [0.65, 0.74]	1	0.43 [0.36, 0.49]	-0.31 [-0.38, -0.23]
$p_{internet}$	0.5 [0.44, 0.56]	0.43 [0.36, 0.49]	1	0.33 [0.26, 0.4]
$p_{Children}$	-0.39 [-0.45, -0.32]	-0.31 [-0.38, -0.23]	0.33 [0.26, 0.4]	1

TABLE S1.

Corrleation (Pearson's ρ) of internet usage with demographic variables
(Greater Sydney, COVID-19 1st wave)

	income security	$p_{WFH}(MTWP)$	$p_{internet}$	$p_{children}$
ds_{baseline}	-0.57 [-0.64, -0.49]	-0.57 [-0.64, -0.49]	-0.081 [-0.19, 0.033]	0.72 [0.66, 0.77]
ds_{peak1}	0.027 [-0.088, 0.14]	-0.12 [-0.24, -0.01]	0.42 [0.32, 0.51]	0.69 [0.62, 0.74]
Δds_{peak1}	0.63 [0.56, 0.7]	0.38 [0.28, 0.47]	0.72 [0.66, 0.77]	0.17 [0.059, 0.28]
$\Delta ds_{\text{peak1}}/ds_{\text{baseline}}$	0.79 [0.74, 0.83]	0.61 [0.54, 0.68]	0.53 [0.44, 0.61]	-0.23 [-0.33, -0.11]
us_{baseline}	0.18 [0.064, 0.29]	0.12 [0.0048, 0.23]	0.17 [0.057, 0.28]	0.012 [-0.1, 0.13]
us_{peak1}	0.81 [0.76, 0.84]	0.5 [0.41, 0.58]	0.55 [0.46, 0.62]	-0.18 [-0.29, -0.066]
Δus_{peak1}	0.78 [0.73, 0.82]	0.47 [0.37, 0.55]	0.53 [0.44, 0.6]	-0.16 [-0.27, -0.05]
$\Delta us_{\text{peak1}}/us_{\text{baseline}}$	0.64 [0.57, 0.7]	0.37 [0.27, 0.47]	0.39 [0.29, 0.48]	-0.19 [-0.3, -0.074]
income security	1	0.65 [0.58, 0.72]	0.58 [0.5, 0.65]	-0.36 [-0.45, -0.25]
$p_{WFH}(MTWP)$	0.65 [0.58, 0.72]	1	0.41 [0.31, 0.5]	-0.31 [-0.41, -0.2]
$p_{internet}$	0.58 [0.5, 0.65]	0.41 [0.31, 0.5]	1	0.27 [0.16, 0.38]
$p_{Children}$	-0.36 [-0.45, -0.25]	-0.31 [-0.41, -0.2]	0.27 [0.16, 0.38]	1

TABLE S2.

Corrleation (Pearson's ρ) of internet usage with demographic variables
(Greater Melbourne, COVID-19 1st wave)

	income security	$p_{WFH}(MTWP)$	$p_{internet}$	$p_{children}$
ds_{baseline}	-0.46 [-0.55, -0.37]	-0.47 [-0.55, -0.37]	0.19 [0.08, 0.3]	0.71 [0.65, 0.76]
ds_{peak1}	0.015 [-0.098, 0.13]	-0.071 [-0.18, 0.043]	0.61 [0.54, 0.68]	0.69 [0.62, 0.74]
Δds_{peak1}	0.55 [0.46, 0.62]	0.48 [0.39, 0.56]	0.7 [0.63, 0.75]	0.24 [0.13, 0.34]
$\Delta ds_{\text{peak1}}/ds_{\text{baseline}}$	0.76 [0.71, 0.8]	0.72 [0.66, 0.77]	0.44 [0.34, 0.53]	-0.3 [-0.4, -0.19]
us_{baseline}	0.04 [-0.074, 0.15]	0.062 [-0.052, 0.17]	0.21 [0.099, 0.32]	0.18 [0.068, 0.29]
us_{peak1}	0.42 [0.32, 0.51]	0.32 [0.21, 0.42]	0.44 [0.35, 0.53]	0.058 [-0.055, 0.17]
Δus_{peak1}	0.4 [0.31, 0.49]	0.31 [0.2, 0.41]	0.38 [0.28, 0.48]	0.0016 [-0.11, 0.11]
$\Delta us_{\text{peak1}}/us_{\text{baseline}}$	0.26 [0.15, 0.36]	0.17 [0.057, 0.28]	0.14 [0.029, 0.25]	-0.1 [-0.21, 0.01]
income security	1	0.72 [0.66, 0.77]	0.42 [0.32, 0.51]	-0.45 [-0.53, -0.35]
$p_{WFH}(MTWP)$	0.72 [0.66, 0.77]	1 [1, 1]	0.41 [0.31, 0.5]	-0.34 [-0.44, -0.24]
$p_{internet}$	0.42 [0.32, 0.51]	0.41 [0.31, 0.5]	1	0.39 [0.29, 0.49]
$p_{Children}$	-0.45 [-0.53, -0.35]	-0.34 [-0.44, -0.24]	0.39 [0.29, 0.49]	1

TABLE S3.

Corrleation (Pearson's ρ) of internet usage with demographic variables
(Greater Sydney, COVID-19 2nd wave)

	income security	$p_{WFH}(MTWP)$	$p_{internet}$	$p_{children}$
ds_{baseline}	-0.57 [-0.64, -0.49]	-0.57 [-0.64, -0.49]	-0.081 [-0.19, 0.033]	0.72 [0.66, 0.77]
ds_{peak2}	-0.085 [-0.2, 0.03]	-0.27 [-0.37, -0.16]	0.33 [0.23, 0.43]	0.71 [0.65, 0.76]
Δds_{peak2}	0.76 [0.71, 0.8]	0.42 [0.32, 0.51]	0.66 [0.59, 0.72]	-0.044 [-0.16, 0.071]
$\Delta ds_{\text{peak2}}/ds_{\text{baseline}}$	0.83 [0.79, 0.86]	0.55 [0.47, 0.63]	0.45 [0.36, 0.54]	-0.32 [-0.42, -0.21]
us_{baseline}	0.18 [0.064, 0.29]	0.12 [0.0048, 0.23]	0.17 [0.057, 0.28]	0.012 [-0.1, 0.13]
us_{peak2}	0.85 [0.81, 0.88]	0.47 [0.37, 0.55]	0.52 [0.43, 0.6]	-0.24 [-0.35, -0.13]
Δus_{peak2}	0.84 [0.8, 0.87]	0.44 [0.35, 0.53]	0.51 [0.42, 0.59]	-0.23 [-0.34, -0.12]
$\Delta us_{\text{peak2}}/us_{\text{baseline}}$	0.74 [0.68, 0.79]	0.38 [0.28, 0.47]	0.42 [0.32, 0.51]	-0.24 [-0.35, -0.13]
income security	1	0.65 [0.58, 0.72]	0.58 [0.5, 0.65]	-0.36 [-0.45, -0.25]
$p_{WFH}(MTWP)$	0.65 [0.58, 0.72]	1	0.41 [0.31, 0.5]	-0.31 [-0.41, -0.2]
$p_{internet}$	0.58 [0.5, 0.65]	0.41 [0.31, 0.5]	1	0.27 [0.16, 0.38]
$p_{Children}$	-0.36 [-0.45, -0.25]	-0.31 [-0.41, -0.2]	0.27 [0.16, 0.38]	1

TABLE S4.

Correlation (Pearson's ρ) of internet usage with demographic variables
(Greater Melbourne, COVID-19 2nd wave)

	income security	$p_{WFH}(MTWP)$	$p_{internet}$	$p_{children}$
ds_{baseline}	-0.46 [-0.55, -0.37]	-0.47 [-0.55, -0.37]	0.19 [0.08, 0.3]	0.71 [0.65, 0.76]
ds_{peak2}	0.0055 [-0.11, 0.12]	-0.0058 [-0.12, 0.11]	0.65 [0.58, 0.71]	0.75 [0.69, 0.79]
Δds_{peak2}	0.35 [0.24, 0.44]	0.38 [0.27, 0.47]	0.74 [0.68, 0.79]	0.49 [0.4, 0.57]
$\Delta ds_{\text{peak2}}/ds_{\text{baseline}}$	0.65 [0.58, 0.71]	0.7 [0.64, 0.75]	0.51 [0.42, 0.59]	-0.091 [-0.2, 0.023]
us_{baseline}	0.04 [-0.074, 0.15]	0.062 [-0.052, 0.17]	0.21 [0.099, 0.32]	0.18 [0.068, 0.29]
us_{peak2}	-0.1 [-0.22, 0.0081]	-0.11 [-0.22, -0.00082]	0.19 [0.074, 0.29]	0.21 [0.099, 0.32]
Δus_{peak2}	-0.11 [-0.22, 0.0032]	-0.12 [-0.23, -0.007]	0.12 [0.0072, 0.23]	0.15 [0.033, 0.25]
$\Delta us_{\text{peak2}}/us_{\text{baseline}}$	-0.068 [-0.18, 0.046]	-0.075 [-0.19, 0.039]	-0.007 [-0.12, 0.11]	0.018 [-0.095, 0.13]
income security	1	0.72 [0.66, 0.77]	0.42 [0.32, 0.51]	-0.45 [-0.53, -0.35]
$p_{WFH}(MTWP)$	0.72 [0.66, 0.77]	1	0.41 [0.31, 0.5]	-0.34 [-0.44, -0.24]
$p_{internet}$	0.42 [0.32, 0.51]	0.41 [0.31, 0.5]	1	0.39 [0.29, 0.49]
$p_{Children}$	-0.45 [-0.53, -0.35]	-0.34 [-0.44, -0.24]	0.39 [0.29, 0.49]	1

TABLE S5.

S3. PRINCIPAL COMPONENT ANALYSIS

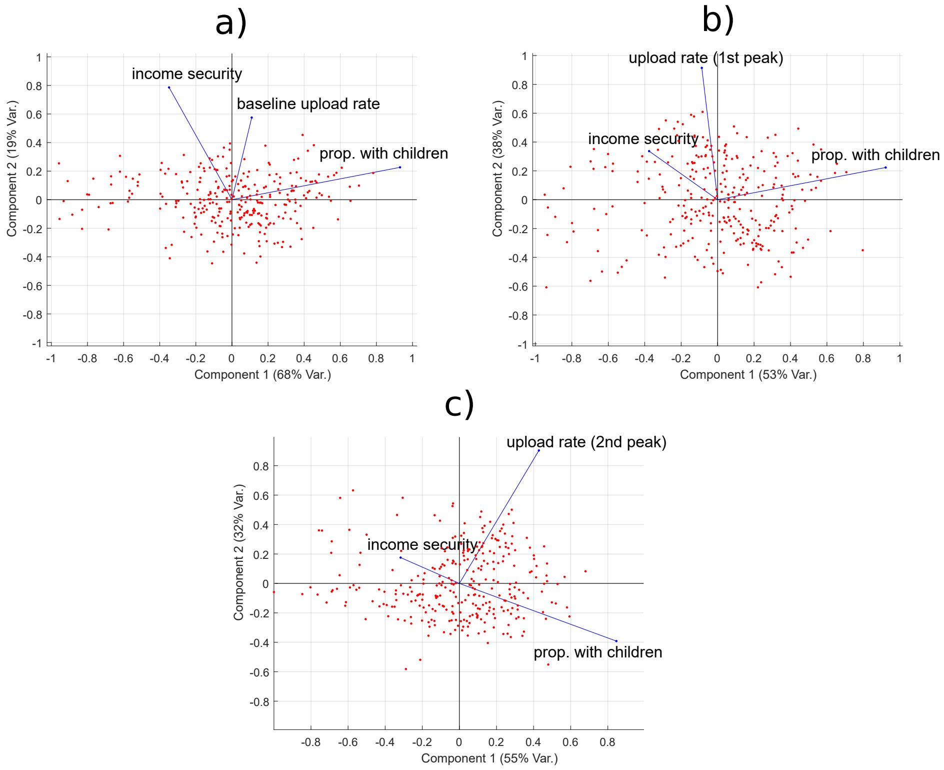

FIG. S2. Biplots demonstrating the results of a 3-component principal component analysis for the SA2 regions of Greater Melbourne, using income security, proportion of families with children, and (a) baseline uploads, (b) absolute upload rates during the first wave of COVID-19 restrictions, and (c) absolute upload rates during the 2nd wave of COVID-19 restrictions. The red dots represent SA2 regions, which are positioned based on the corresponding values of the first two principal components. The blue vector lines represent the contributions of each variable (labeled) to these two components.

S4. DISTRIBUTIONS OF INCOME SECURITY AND WORK-FROM-HOME CLASSIFICATION BY OCCUPATION AND SA2 REGION

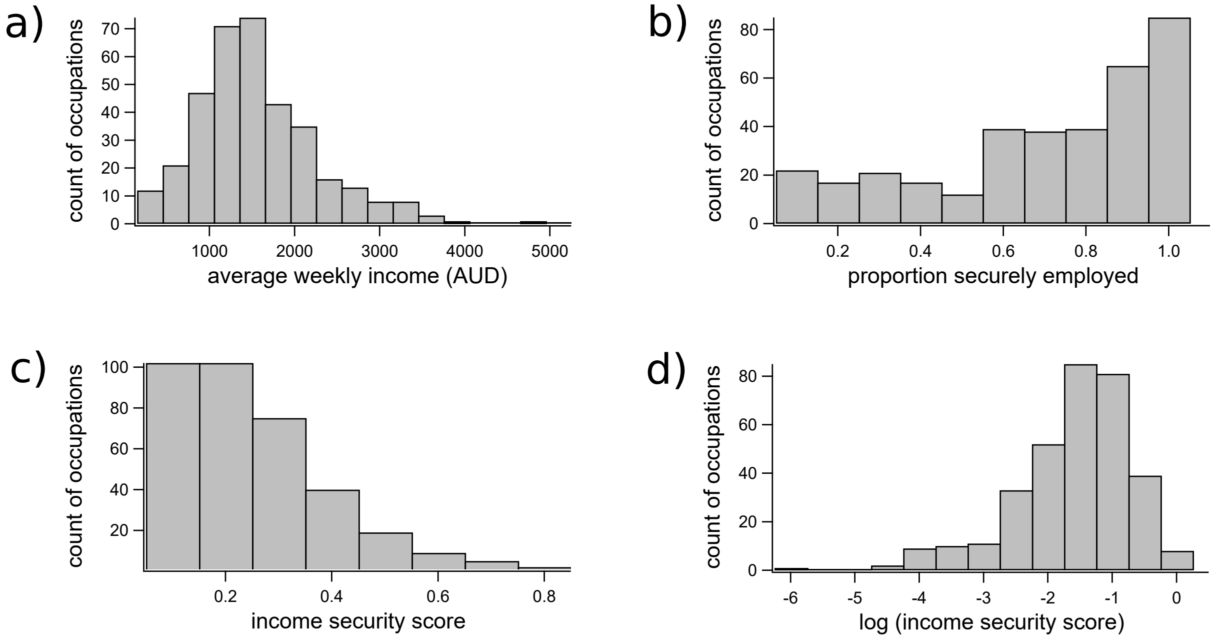

FIG. S3. Histograms demonstrating the distributions among occupation classifications of (a) income, (b) proportion securely employed, (c) income security scores computed as the product of relative income and proportion securely employed, and (d) log-transformed income security scores.

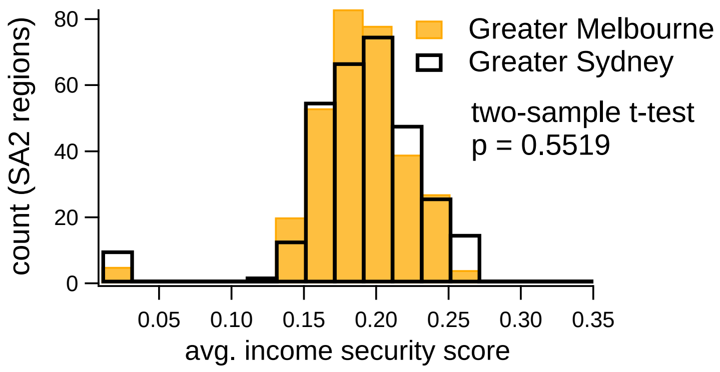

FIG. S4. Histograms demonstrating the distribution of average income security scores over the SA2 regions in Greater Melbourne (solid yellow bars) and Greater Sydney (open black bars).

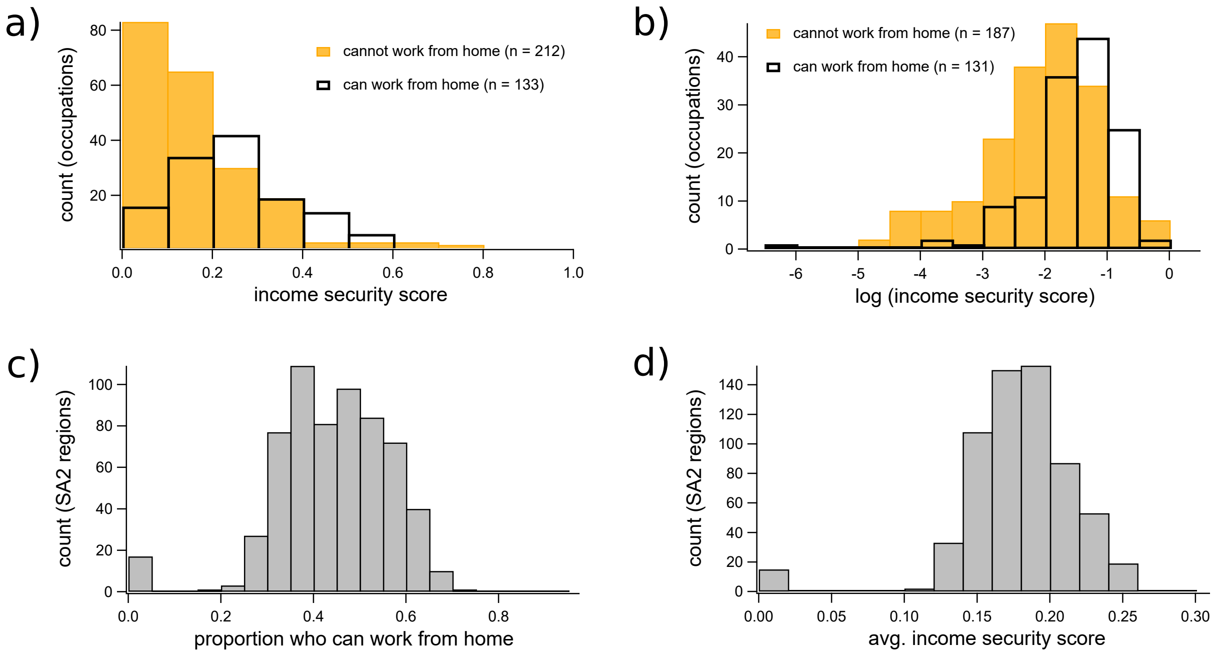

FIG. S5. (a, b) Histograms demonstrating the distribution of (a) income security by occupation, grouped by the ability to work from home, with the log-transformed distributions shown in (b). In (a) and (b), open black bars represent occupations for which at least 50% of HILDA respondents were securely employed, while yellow bars represent those occupations for which less than 50% of HILDA respondents were securely employed. (c, d) Histograms showing the distribution among SA2 regions of (c) the proportion of occupied individuals who can work from home, and (d) average income security. The histograms in (c) and (d) include all SA2 regions in Greater Sydney and Greater Melbourne.

S5. GEOSPATIAL DISTRIBUTION OF INCOME SECURITY AND CHANGES TO INTERNET ACTIVITY

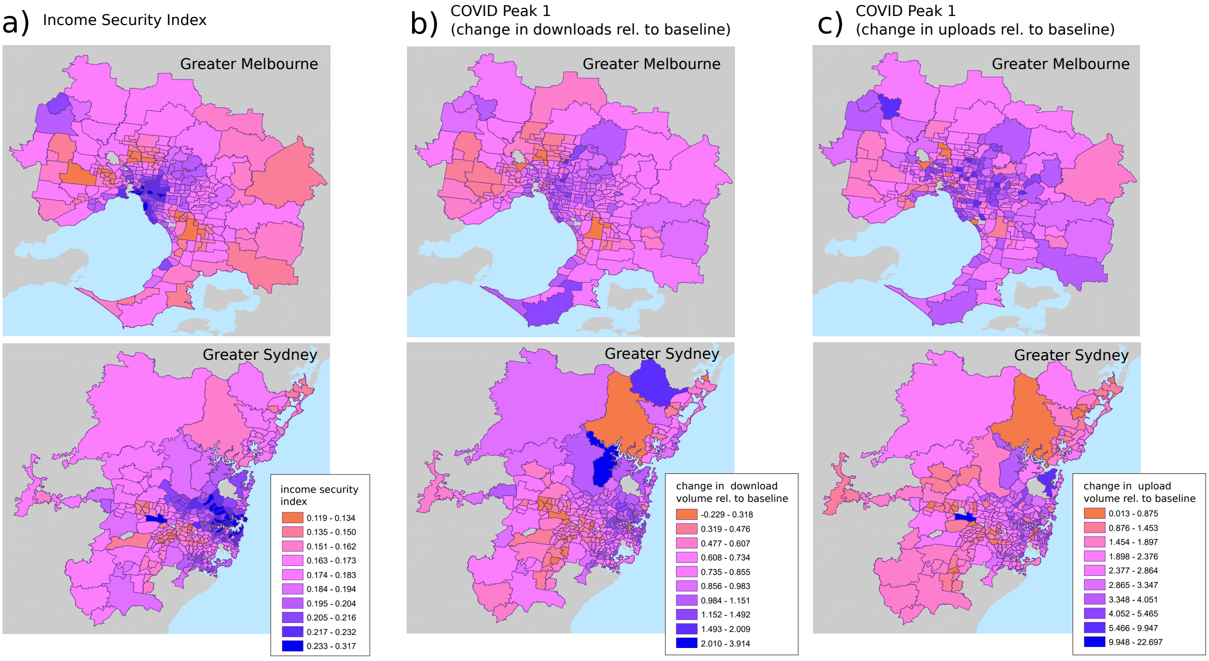

FIG. S6. Choropleth maps demonstrating the geospatial distributions of income security and changes to internet activity during the first wave of COVID-19 restrictions in Greater Sydney and Greater Melbourne. The maps in (a) show the spatial distribution of aggregate income security, while (b) and (c) show the spatial distribution of changes in download and upload volumes (respectively) during the first-wave period (April 18th to April 24th, 2020).

S6. TESTS OF GEOSPATIAL AUTOCORRELATION OF INCOME SECURITY AND CHANGES TO INTERNET ACTIVITY

We have computed the Global Moran's I statistics of income security by SA2, as well as relative changes to internet use at Peak 1 and Peak 2 in both Melbourne and Sydney.

The Moran's I statistic is a descriptor of the extent to which a variable is spatially autocorrelated, or, conversely, whether the phenomenon studied is randomly distributed in space (which is typically the null hypothesis).

The Moran's I statistics for both Melbourne and Sydney are shown in Table S6. All statistics have been computed with the assumption of a `Queen` neighbourhood, with a row-standardised weight distribution between neighbours, using the permutation-based Moran's I statistic implementation in the `spdep` package [1, 2] of the R statistical environment, based on 1000 Monte-Carlo runs.

TABLE S6. Caption

City	Variable	Moran's I	p-value
Melbourne	Income security	0.736	$< .001$
	Peak 1 (US)	0.251	$< .001$
	Peak 2 (US)	0.131	$< .001$
	Peak 1 (DS)	0.574	$< .001$
	Peak 2 (DS)	0.501	$< .001$
Sydney	Income security	0.786	$< .001$
	Peak 1 (US)	0.510	$< .001$
	Peak 2 (US)	0.656	$< .001$
	Peak 1 (DS)	0.596	$< .001$
	Peak 2 (DS)	0.588	$< .001$

Changes to internet use relative to baseline show higher levels of autocorrelation for downloads than for uploads at both peaks in Melbourne (Figure S7(b,c,d,e)), while in Sydney these correlations remain the same (Figure S9(b,c,d,e)). Yet, the permutation-based Moran's tests confirm that all systems are significantly autocorrelated, as illustrated by the permutation-based density plots in Figures S8 and S10).

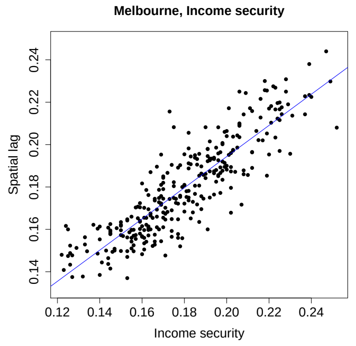

(a) Income security

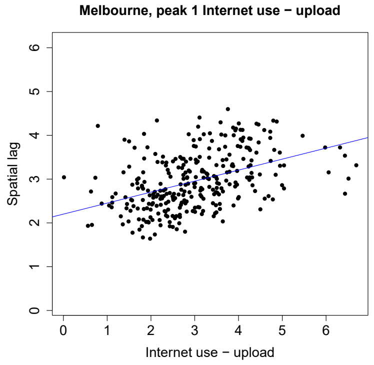

(b) Internet use - Peak 1 (US), change relative to baseline

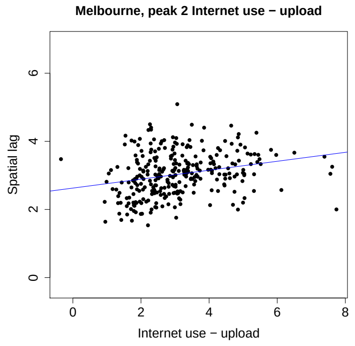

(c) Internet use - Peak 2 (US), change relative to baseline

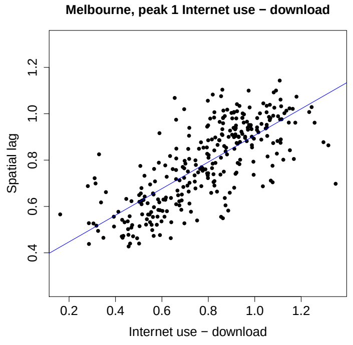

(d) Internet use - Peak 1 (DS), change relative to baseline

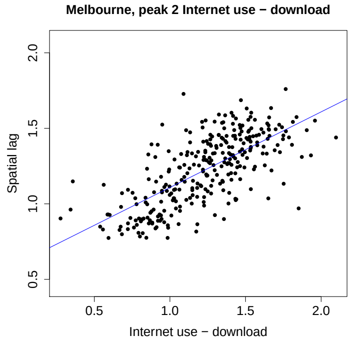

(e) Internet use - Peak 2 (DS), change relative to baseline

FIG. S7. Melbourne - Plots of the variables and their spatially lagged values for income security (a), as well as relative change to upload volume at peak 1 (b), relative change to upload volume at peak 2 (c), relative change to download volume at peak 1 (d), and relative change to download volume at peak 2 (e).

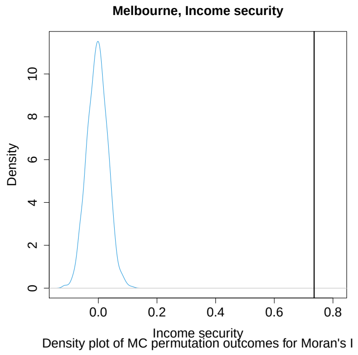

(a) Income security

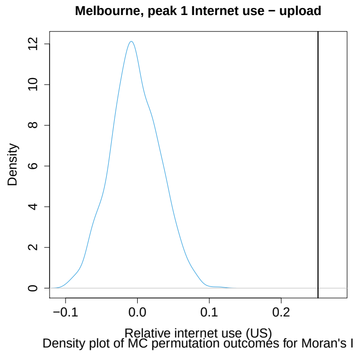

(b) Internet use - Peak 1 (US), change relative to baseline

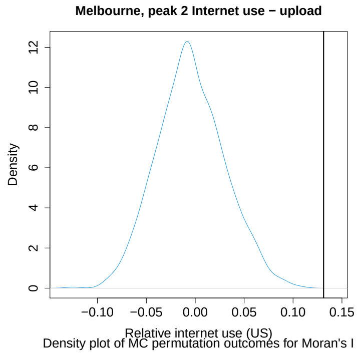

(c) Internet use - Peak 2 (US), change relative to baseline

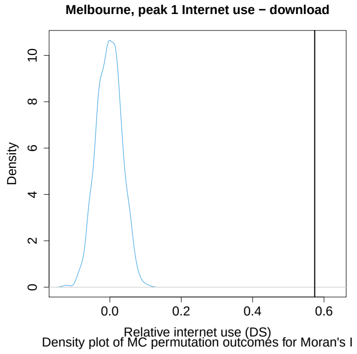

(d) Internet use - Peak 1 (DS), change relative to baseline

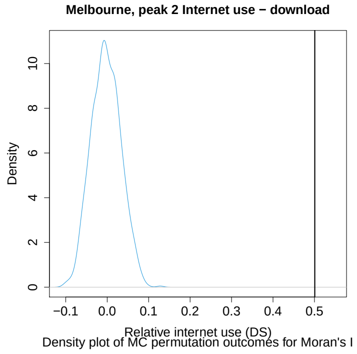

(e) Internet use - Peak 2 (DS), change relative to baseline

FIG. S8. Melbourne - density plots of Moran's I permutations to complement the tests of statistical significance of Moran's I statistics for income security (a), as well as relative change to upload volume at peak 1 (b), relative change to upload volume at peak 2 (c), relative change to download volume at peak 1 (d), and relative change to download volume at peak 2 (e).

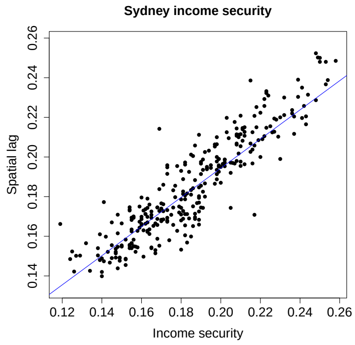

(a) Income security

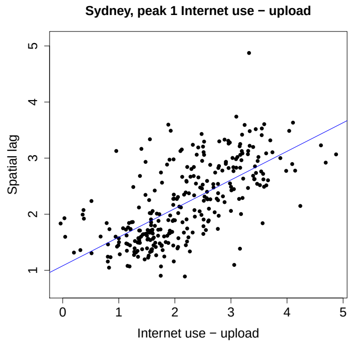

(b) Internet use - Peak 1 (US), change relative to baseline

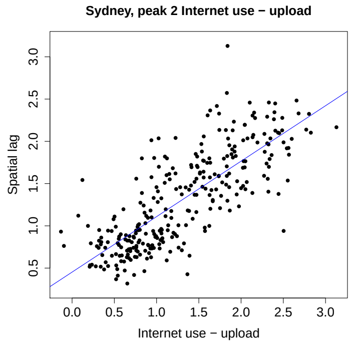

(c) Internet use - Peak 2 (US), change relative to baseline

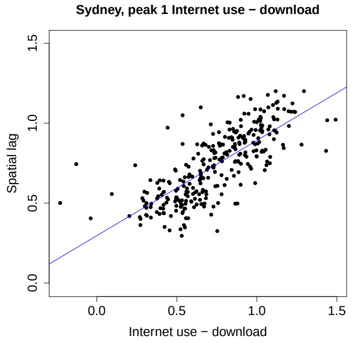

(d) Internet use - Peak 1 (DS), change relative to baseline

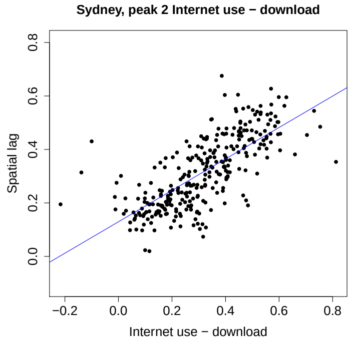

(e) Internet use - Peak 2 (DS), change relative to baseline

FIG. S9. Sydney - Plots of the variables and their spatially lagged values for income security (a), as well as relative change to upload volume at peak 1 (b), relative change to upload volume at peak 2 (c), relative change to download volume at peak 1 (d), and relative change to download volume at peak 2 (e).

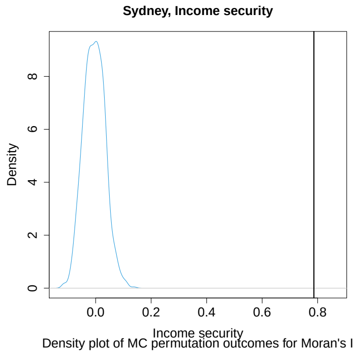

(a) Income security

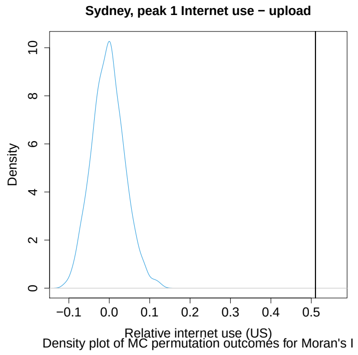

(b) Internet use - Peak 1 (US), change relative to baseline

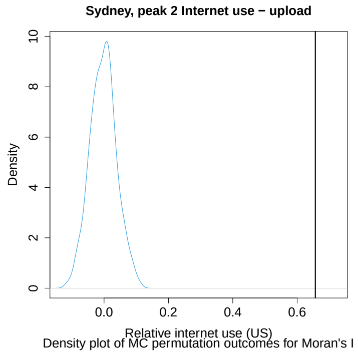

(c) Internet use - Peak 2 (US), change relative to baseline

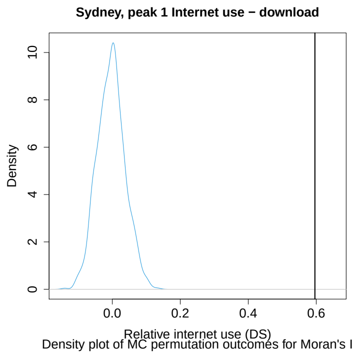

(d) Internet use - Peak 1 (DS), change relative to baseline

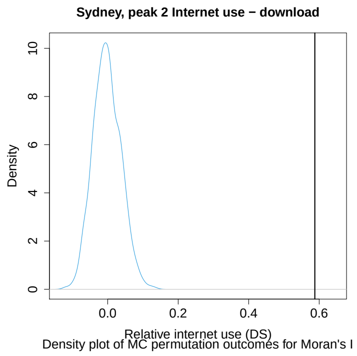

(e) Internet use - Peak 2 (DS), change relative to baseline

FIG. S10. Sydney - Density plots of Moran's I permutations, complementing tests of statistical significance of Moran's I statistics for income security (a), as well as relative change to upload volume at peak 1 (b), relative change to upload volume at peak 2 (c), relative change to download volume at peak 1 (d), and relative change to download volume at peak 2 (e).

S7. REFERENCES

[1] Bivand RS, Pebesma E, Gomez-Rubio V. Applied spatial data analysis with R, Second edition. Springer, NY; 2013. Available from: https://asdar-book.org/.

[2] Bivand R, Wong DWS. Comparing implementations of global and local indicators of spatial association. TEST. 2018;27(3):716–748. Available from: https://doi.org/10.1007/s11749-018-0599-x.
